# An expression database for roots of the model legume *Medicago truncatula *under salt stress

**DOI:** 10.1186/1471-2164-10-517

**Published:** 2009-11-11

**Authors:** Daofeng Li, Zhen Su, Jiangli Dong, Tao Wang

**Affiliations:** 1 State Key Laboratory of Agrobiotechnology, College of Biological Sciences, China Agricultural University, Beijing, 100193, PR China; 2 State Key Laboratory of Plant Physiology and Biochemistry, College of Biological Sciences, China Agricultural University, Beijing 100193, PR China

## Abstract

**Background:**

*Medicago truncatula *is a model legume whose genome is currently being sequenced by an international consortium. Abiotic stresses such as salt stress limit plant growth and crop productivity, including those of legumes. We anticipate that studies on *M. truncatula *will shed light on other economically important legumes across the world. Here, we report the development of a database called MtED that contains gene expression profiles of the roots of *M. truncatula *based on time-course salt stress experiments using the Affymetrix Medicago GeneChip. Our hope is that MtED will provide information to assist in improving abiotic stress resistance in legumes.

**Description:**

The results of our microarray experiment with roots of *M. truncatula *under 180 mM sodium chloride were deposited in the MtED database. Additionally, sequence and annotation information regarding microarray probe sets were included. MtED provides functional category analysis based on Gene and GeneBins Ontology, and other Web-based tools for querying and retrieving query results, browsing pathways and transcription factor families, showing metabolic maps, and comparing and visualizing expression profiles. Utilities like mapping probe sets to genome of *M. truncatula *and In-Silico PCR were implemented by BLAT software suite, which were also available through MtED database.

**Conclusion:**

MtED was built in the PHP script language and as a MySQL relational database system on a Linux server. It has an integrated Web interface, which facilitates ready examination and interpretation of the results of microarray experiments. It is intended to help in selecting gene markers to improve abiotic stress resistance in legumes. MtED is available at http://bioinformatics.cau.edu.cn/MtED/.

## Background

Legumes are important economic crops that provide humans with food, livestock with feed, and industry with raw materials [1]. Additionally, legumes can fix nitrogen with rhizobia in soil, and the plants do not require external nitrogen sources such as nitrogen fertilizers [2]. *Medicago truncatula *(the barrel medic) has been selected as a model legume because it is self-fertile and diploid, and has a short life cycle and a relatively small genome [3-5]. The genome of the cultivar Jemalong line A17 is currently being sequenced by the Medicago Genome Sequencing Consortium (MGSC) [6].

Plant growth and crop productivity is largely limited by environmental factors that include water-deficit stress, such as by salinity, which bring about large-scale alterations in gene expression in plants [7]. High NaCl concentrations in soil cause salinity stress and limit the geographical distribution of most highly salinity-sensitive plants, including legumes [8,9]. Plant responses and adaptations to abiotic stresses are complex [10], and include signaling and transcription regulatory processes that are interrupted and reestablished to provide homeostasis [11], necessitating whole-genome studies of stress-resistance mechanisms. Recently, the rapid development of microarray technology has provided a powerful tool for genome-scale gene expression analyses [12-14].

Previous studies on *M. truncatula *showed differential adaptation and expression of the TFIIIA regulatory pathway in response to salt stress between two genotypes, 108-R and Jemalong A17 [15,16]. We used the Affymetrix GeneChip Medicago Genome Array to analyze the time-course transcriptome profile of the roots of *M. truncatula *genotype Jemalong line A17 seedlings at young seedlings development periods under 180 mM concentrations of NaCl at different salt treatment time points, and we constructed a database, MtED, to store and provide the results through a friendly Web interface. To our knowledge, this work is unique in the field of legume salt stress responses. We sought to identify genes from the model legume *M. truncatula *that may function in salt stress resistance.

Public resources like MtGEA [17] (*Medicago truncatula *Gene Expression Atlas) and LIS [18] (Legume Information System) also contain transcriptome data of *Medicago truncatula*. MtGEA currently mainly focuses on detailed developmental time-series gene expression profiles of major organ systems (roots, stems, and leaves etc.) of *Medicago truncatula*. LIS places extra emphasis on cross-species comparison of molecular and genetic data from legumes include *Medicago truncatula, Lotus japonicus, Glycine max*, and also *Arabidopsis thaliana*. Our database MtED contains expression profiles of roots at young seedling developmental stage of *Medicago truncatula *under 180 mM concentration of salt stress. Annotations of probe sets in MtED include details of BLAST matched Tentative Consensus sequences (TCs), proteins of *Medicago truncatula *and *Arabidopsis thaliana*. Transcription factor family assignment and chromosome location are also provided. Web interface of MtED is easy to use and additionally provides function category analysis based on Gene Ontology and GeneBins Ontology, respectively.

## Construction and content

### Microarray experiment design

Salinity in soil affects the roots of plants first. We chose to study the roots at young seedlings development periods as samples for our microarray experiments. We placed germinated seedlings in Petri dishes, using 180 mM NaCl for the salt treatment and collected roots at 0, 6, 24, and 48 hours after beginning the stress, three biological replicates of each time point. Total RNA was isolated from roots of young seedlings using a reagent kit (Autolab, http://www.autolabtech.cn/, Beijing, China), performed according to the manufacture's protocol. We use the Affymetrix GeneChip Hybridization Oven 640 to perform hybridization at 45°C with rotation for 16 h, and the hybridization data were analyzed using GeneChip Operating software (GCOS 1.4) using the default setting for generating raw data (CEL file) files. Data of our microarray experiments have been deposited in NCBI's Gene Expression Omnibus [19] and are accessible through GEO Series number GSE13921 http://www.ncbi.nlm.nih.gov/geo/query/acc.cgi?acc=GSE13921).

### Data sources

The consensus sequences of the probe sets on the GeneChip Medicago Genome Array were from Affymetrix. Tentative Consensus sequences (TCs) of *M. truncatula *come from the DFCI Medicago Gene Index project [20] and their annotations. Protein sequences and their annotations were derived from the Medicago Genome Sequence Consortium (MGSC) project [6]. Protein sequences of *Arabidopsis thaliana *and their annotations came from the TAIR database, version 9 [21].

Pathway information came from GeneBins [22], in GeneBins format. We downloaded MapMan-formatted [23] results via the GeneBins Website [24]. Metabolic map information links were obtained from the KEGG *Medicago truncatula *EST database [25]. Transcriptional profiles of *M. truncatula *responses to 180 mM concentration of NaCl and expression data at different time points were determined using the Affymetrix GeneChip Medicago Genome Array.

### Probe set annotation procedure and GO annotation

The consensus sequence of each probe set was provided by Affymetrix [26]. First, we used the consensus sequence to conduct a BLASTN [27] search with *M. truncatula*'s TCs, provided by the DFCI Gene Index project. Then, we used the consensus sequences to perform a BLASTX [27] search with *M. truncatula *protein sequences, provided by the Medicago Genome Sequence Consortium (MGSC), and *A. thaliana *proteins (TAIR 9 version), respectively. The annotation of the best match was assigned to the probe set (best BLAST hit method) [28]. We set the e-value cut-off to be 10^-5 ^and the length of the HSP (high-scoring segment pair) to be longer than 100 bp when we used Perl http://www.perl.org/ and BioPerl [29] scripts to analyze the BLAST search results. 49684, 38728 and 33063 unique probe sets of all 50902 *Medicago truncatula *probe sets had been annotated. Only top 5 homologs were selected for easily storage if more than 5 homologs found to meet the selection criteria above. All matching information for each probe set was imported to the MtED database to facilitate Web searches and displays.

Gene Ontology (GO) annotation of all *M. truncatula *probe sets consists of two parts. First part annotation was obtained from EasyGO [30], which generated GO annotations for 18248 unique *M. truncatula *probe sets by InterProScan [31,32]. Second part annotation was obtained from TC homologs, which annotate 2501 unique *M. truncatula *probe sets. Total 20749 *M. truncatula *probe sets were annotated by Gene Ontology finally.

### Transcription factors

Consensus sequences of all *Medicago truncatula *probe sets were used to conduct a BLASTX search against *Medicago truncatula *transcription factor peptide sequences provided by PlantTFDB database [33]. We found 2138 probe sets to have sequence similarity to those 1022 transcription factors under E-value cut-off as 10^-5 ^and length of HSP longer than 100 bp. Information of probe sets assign to which transcription factor family were also stored and can be viewed through our database.

### Microarray data analysis

We use GeneChip operating software (GCOS 1.4) to analyze the hybridization data and generate raw data (CEL file) files using the default settings. The affylmGUI [34] package in R [35] and BioConductor [36] environment was used for further data analysis. The GCRMA [37] algorithm was used to normalize the raw data during the analysis process. The log_2_-transformed expression values and the corresponding statistic p-values were exported as a tab-delimited file and then imported to a local database.

### Expression profile assignment

A tab-delimited file contained expression value of all *Medicago truncatula *probe sets was used as input of STEM software [38] for clustering and visualizing expression profile using default settings. Parts of results of STEM were deposited in and can be viewed through our database.

### Database schema and implementation

All MtED data are stored in a MySQL http://www.mysql.com/ relational database called MtED. It consists of 18 tables and its architecture is shown in Additional File 1. Core part is the *experiment *table stores expression value of our experiment, annotation part consists of 5 tables contains BLAST homologs of probe sets, sequence part consists of 5 tables contains sequences of corresponding homologs, additionally 2 tables contain GO annotation and 3 tables contain pathway information, and table *STEM_profile *and *blat *stores information of expression profile and genome locations of probe sets, respectively.

## Utility and discussion

### Web interface overview

The Web interface of MtED is written in PHP http://www.php.net/ scripting language. The home page briefly introduces MtED's content and our microarray experiments. Annotation information for each probe set can be sought out through the search page, and sequences of probe sets or proteins appearing on the search result page can be displayed through hypertext links. The search page also provides GO annotation and expression data batch search function, and a GO search results page provides GO functional category analysis and displays three histograms under biological processes, molecular function, and cellular component levels providing the functional distribution of the inputted probe set list. In addition, probe sets contained in each functional category can be viewed through a table below these histograms, and red background row means statistic significant. The browse pathway page shows hierarchical pathways based on GeneBins Ontology. Each pathway shows the corresponding probe set list annotated for this pathway. Probe sets can also be searched on this page; the search result shows the parent pathways of the pathway to which the input probe set belongs, and one probe set may belong to more than one pathway. The browse transcription factor family page shows number of probe sets assigned to which transcription factor family, clicking each family name will show all probe sets belong to this family. The browse expression profile page shows expression profile generated by STEM software which named from *a *to *k*, clicking each profile name also shows probe sets belong to this profile. The experiment page shows the results of our microarray experiment. In comparison to the expression value at time point 0 h, log_2_-transformed expression values at each other time point and each probe set are shown on this page. Value in red background cell means its p-value less than 0.05. The upwards and downwards arrows at each time point provide a sort function according to the expression value. Probe sets also can be searched on this page. The detail button on the right of each row links to a page detailing the results of the experiment, with expression curve graphs and other annotation links. The KEGG pathway page provides information obtained from the KEGG[39,40]*Medicago truncatula *EST database[25,41]. The searched probe set entered on this page will return emtr homologs (*Medicago truncatula *ESTs data)[25,41] containing brief information about the BLASTN result, and pathways that the emtr belongs to are also provided through clickable buttons below. Clicking a button below will also link to the corresponding page in the KEGG database, showing a metabolic pathway map and in which the emtr was marked in a red rectangle.

### Functional category analysis

Recently, the Gene Ontology (GO) annotation system [42] has become widely used in many fields, including the interpretation of microarray data [43]. MtED also provides GO functional category analysis of a list of *M. truncatula *probe sets. Users can enter a list of probe sets on the search page then press the Search GO Info button, and the results page will return the GO terms annotated to each probe set and the annotation source from EasyGO or *M. truncatula *TC homologs. The search results page above supplies a form that can provide GO functional category analysis, as mentioned above. This function was achieved via PHP scripts and MySQL storied Gene Ontology termdb database, released by the Gene Ontology database [44]. The dynamic graph was drawn using the Open Flash Chart software [45]. In addition, GeneBins [22] ontology was used in some recent studies about transcriptome analysis of *Medicago truncatula *[46,47]. Thus, functional distribution of a list of probe sets in the second level of the GeneBins ontology was also adopted in our database, which is similar to that provided by GeneBins. Statistic analysis was implemented during both functional category analyses by R [35] tasks running in background. Whether a probe set could annotated to a functional category can be regarded as a Bernolli trial [30], and the probability of a probe set successfully annotated to a functional category can be calculated as number of probe sets annotated to this category divided by number of total probe sets in the microarray which had been annotated. So we can calculate a P-value for each functional category using binomial test based on the following three numbers: number of probe sets annotated to this functional category in user-input list, number of user-input list of probe sets and the probability of the functional category been annotated by a probe set. For controlling false positive rate, FDR adjusted P-values were calculated by a false discovery rate (FDR) [48] correction test based on P-values of binomial test. Result page of both category enrichment analysis also contains a table summarize the distribution of inputted list of probe sets, red background rows means FDR-adjusted P-value less than selected p-value cut-off. User can change p-value cut-off in result page and can sort the table by FDR-adjusted P-value or Number of probe sets submitted column, note it may lag the browse for a little while when sort a big table.

**Figure 1:**
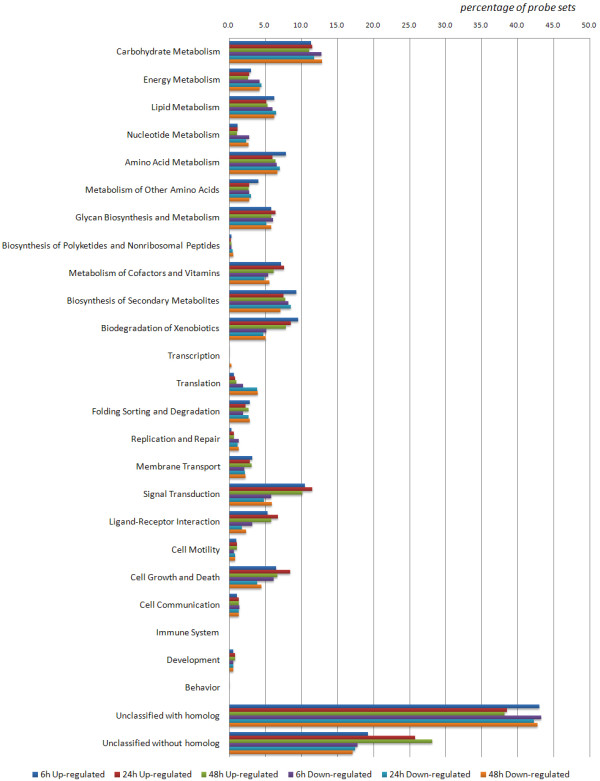
**Functional category enrichment analysis of differentially expressed probe sets at different time points based on GeneBins Ontology**. Up-regulated or Down-regulated means expression of probe sets at one time point changed more or less than 2 fold versus 0 hour, respectively, and p-value < 0.05.

**Figure 2:**
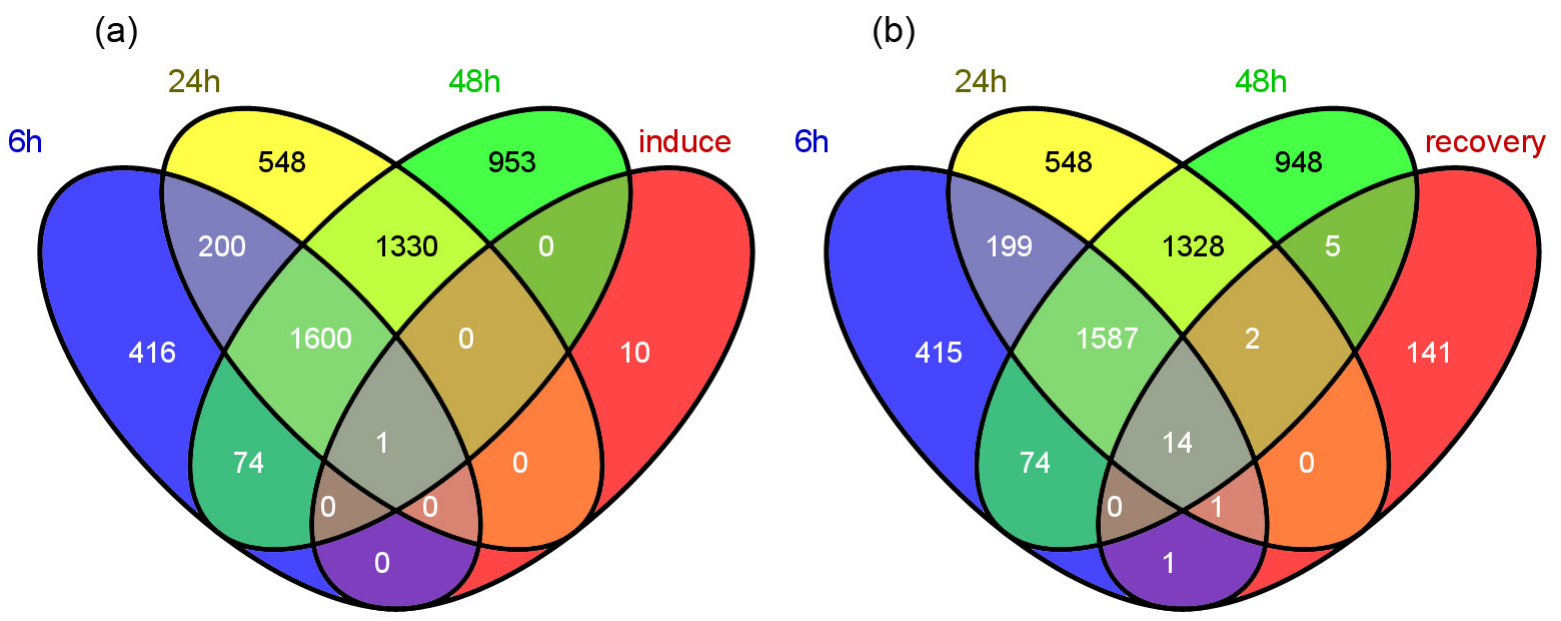
**Overlap of early-induced (a) and recovery-related (b) genes in a previous report between probe sets that significant up-regulate more than 2 fold at each time point**. Note: the numbers of probe sets identified as early-induced and recovery-related after salt stress in our work were different from the numbers reported in the previous study; this is because the version of the *Medicago truncatula *sequence release has been changed.

Microarray technology has been used in many plants, and function as a powerful tool for high-thought screening of genes responsive to different abiotic stresses [49-54]. A number of studies focus on gene expression patterns in response to salt stress were monitored in *Arabidopsis *[13,55] and rice [50,56-59] as their whole-genome sequence released earlier [60,61]. Based on those studies, it is known that a number of genes show dynamic expression changes at a genomic-level scale while response to salt stress. Those genes identified as salt-responsive genes are classified to different groups according to their putative functions. These groups are partly same among different plants mainly include primary energy metabolism, protein metabolism, cell walls, transporters, carbohydrates and etc [55]. In data of our experiment, functional category enrichment analysis of differentially expressed probe sets at different time points based on GeneBins Ontology was presented in Figure 1. Up-regulated or Down-regulated means expression of probe sets at one time point changed more or less than 2 fold versus 0 hour, respectively, and p-value < 0.05. These functional categories are partly same as mentioned above. In a recent study about effects of salt stress on *Medicago truncatula*, 10 early-induced and 156 recovery-related genes in roots after salt stress were identified [62]. Overlap of these genes between probe sets that significant up-regulate more than 2 fold at each time point was explained by Venn diagram by Venny [63] software as shown in Figure 2. Common element in "6 h", "24 h", "48 h" and "induce" (Figure 2a) was probe set Mtr.8498.1.S1_s_at, which has been annotated as an asparagine synthetase. It looks asparagine synthetase gene in *Medicago truncatula *not only function in an early-responsive mode, but also have a long term responsive mode to salt stress in our experiment. The asparagine synthetase gene in alfalfa (*Medicago sativa*) showed a high expression in root nodules and dark-adapted leaves [64]. In other plants like wheat [65] and sunflower [66], expression of asparagine synthetase gene has also been observed under various environmental stresses. In the meanwhile, expression of other probe sets involved in the same metabolic pathway of L-Glutamine transforming to L-Glutamate were also checked, only Mtr.12432.1.S1_at (annotated as NADH-dependent glutamate synthase) showed significant up-regulate more than 2 fold at all time points. Common elements in "6 h", "24 h", "48 h" and "recovery" (Figure 2b) include probes sets annotated as cold and drought-regulated protein (CORA, Mtr.48778.1.S1_x_at), E-class P450 protein (CYP71A25, Mtr.10175.1.S1_at), osmotin-like protein precursor (Mtr.40555.1.S1_at), chitinase (Mtr.331.1.S1_at), protein kinase C and phorbol ester/diacylglycerol binding (Mtr.42918.1.S1_at), ACC oxidase (Mtr.25557.1.S1_at), Haem peroxidise (Mtr.38635.1.S1_at), Zinc finger (Mtr.42911.1.S1_at and Mtr.8696.1.S1_at), asparagine synthase (Mtr.8498.1.S1_s_at), STZ (salt tolerance zinc finger, Mtr.37495.1.S1_at), Pprg2 protein (Mtr.10317.1.S1_at), Ca^2+^-dependent nuclease (Mtr.10781.1.S1_at) and MYB family transcription factor (Mtr.44962.1.S1_at). GO functional category enrichment analysis result of these probe sets through our database was presented in Additional File 2. Categories were sorted by FDR-adjusted P-value and only significant categories were showed (p-value < 0.05). Categories like "response to stimulus" and "response to stress" were enriched most probe sets, illustrating those recovery-related probe sets also contained a long term response after salt stress. Category "response to salt" was also showed but without significance (data not shown).

### Transcriptional factor family distributions

Many transcription factors (TF) have been identified can improve stress tolerance [67-69]. For example, an AP2-EREBP TF family member DREB1, whose over-expression in many plants like Arabidopsis [70] and rice [71,72] improved salt tolerance. SNAC1, which belongs to NAC TF family, make rice more tolerant to salt and drought [73]. Transferring an R1R2R3 type MYB TF isolated from rice to Arabidopsis showed more salt resistance [74]. A transcription factor named AtNAC2 in Arabidopsis is involved in salt stress response [75]. Many members of WRKY TF family are involved in salt stress induction in Arabidopsis [55]. We calculated the transcriptional factor family distributions of probe sets that significant up-regulate more than 2 fold at each time point. Results were illustrated in detail by Table 1. Probe sets belong to WRKY, AP2-EREBP and MYB etc. TF family were indeed induced in our experiment.

**Table 1:** A transcriptional factor family distribution of probe sets that significant up-regulated more than 2 fold at each time point.

Number of probe sets				
**TF Family**	**On array**	**>2 fold up-regulated versus 0 h**		

		**6 h**	**24 h**	**48 h**

ABI3-VP1	53	0	2	3
Alfin	9	0	0	0
AP2-EREBP	152	45	52	50
ARF	36	0	0	0
ARID	12	0	2	1
AS2	28	8	6	6
AUX-IAA	34	5	4	2
BBR-BPC	2	0	0	0
BES1	10	1	2	1
bHLH	144	13	18	15
bZIP	92	7	11	15
C2C2-CO-like	57	15	9	11
C2C2-Dof	34	3	6	6
C2C2-GATA	47	1	0	2
C2C2-YABBY	7	0	0	0
C2H2	86	12	19	20
C3H	162	3	6	8
CAMTA	6	2	2	3
CCAAT-Dr1	7	1	1	0
CCAAT-HAP2	14	1	4	4
CCAAT-HAP3	11	0	0	3
CCAAT-HAP5	15	0	0	1
CPP	4	1	1	0
E2F-DP	10	0	0	2
EIL	7	0	0	0
FHA	6	0	0	0
GARP-ARR-B	12	1	1	1
GARP-G2-like	47	4	2	3
GeBP	3	0	0	0
GIF	3	1	0	1
GRAS	74	11	19	19
GRF	10	0	1	1
HB	76	10	13	14
HMG	15	0	0	0
HSF	27	7	10	11
JUMONJI	16	0	1	2
LFY	1	0	0	0
LIM	19	0	0	0
LUG	66	2	2	2
MADS	49	0	1	1
MBF1	7	0	0	0
MYB	95	15	21	21
MYB-related	116	5	14	13
NAC	88	18	29	36
PcG	33	1	3	2
PHD	71	2	5	8
PLATZ	9	0	1	1
S1Fa-like	4	1	1	1
SBP	19	1	0	0
SRS	8	0	0	0
TAZ	14	2	2	2
TCP	26	2	2	3
TLP	22	0	1	2
Trihelix	22	1	2	2
ULT	1	0	0	0
VOZ	1	0	0	0
Whirly	5	0	0	0
WRKY	95	46	57	55
ZF-HD	11	0	1	1
ZIM	28	13	13	11

### Mapping probe sets to genome

Genome sequence of *Medicago truncatula *are obtained from 3.0 assembly release provided by Medicago Genome Sequence Consortium (MGSC). Consensus sequences of all *Medicago truncatula *probe sets were used to align to the genome sequence by BLAT [76]. Raw results of BLAT generated were filtered by Perl scripts setting identity and coverage cut-off to 90% and 80%, respectively. Filtered result contains 38400 unique probe sets mapping to all *Medicago truncatula *chromosomes. 9594, 9915, 11484, 11601, 11957, 7237, 10746, 10827 unique probe sets were mapped to chromosome 1 to 8, respectively. User can search interested probe set and see detail alignment through our web interface. User can also submit other sequence to see whether and where it can map to any *Medicago truncatula *chromosomes; this util was implemented by webBlat program. User can also submit a pair of primer to see whether it would amplify a target in the genome, and this function was implemented by isPcr which belong to UCSC Genome Browser software suite [77].

### Outer links to the *Medicago truncatula* Gene Expression Atlas

The *Medicago truncatula *Gene Expression Atlas (MtGEA) [17] is a comprehensive platform that provides complete transcriptome profiles of all the major organ systems of *M. truncatula*. We included suitable links on the result pages, search page, and experiment detail page, so that users can readily examine transcriptome information for their probe set of interest in the MtGEA.

## Future prospects

As the *M. truncatula *genome is currently being sequenced by an international consortium, available information on this model legume (including sequences, GO annotation, pathway information) will become more comprehensive and accurate. We will update annotation contents (mainly including GO annotations of TCs and probe sets, GeneBins pathway information, KEGG emtr information) and genome information in MtED with the latest information from the science community.

## Conclusion

We built a local database called MtED that was constructed in the PHP scripting language as a MySQL relational database system based on a Linux server. MtED collects sequences, GO annotation, and pathway information for *M. truncatula*'s sequences, such as probe sets, TCs, and emtrs. The results of our salt stress-treatment microarray experiments are also stored in MtED and users can easily browse the results through MtED's user-friendly Web interface. MtED provides GO functional categories and second GeneBins ontology functional distribution information for a list of probe set. MtED also provides location information of probe sets in the genome, with standalone interface of webBlat for mapping any sequence to genome and webPcr for In-silicon PCR. MtED is freely and fully available at http://bioinformatics.cau.edu.cn/MtED/.

## Availability and requirements

The database is available at http://bioinformatics.cau.edu.cn/MtED/ and is usable with most modern Web browsers. The user's browser must have JavaScript enabled to show query examples and Cookie and Flash to show the expression curves drawn by the Open Flash Chart software.

## Authors' contributions

DL have constructed the database and drafted the manuscript. ZS, JD and TW supervised the project. All authors read and approved the final manuscript.

## Supplementary Material

Additional file 1 **Schema of MtED database**. Schema of MtED database. Click here for file

Additional file 2 **GO functional category analysis of probe sets contained in "6 h", "24 h", "48 h" and "recovery" simultaneously**. Category was sorted by FDR-adjusted P-value. Only significant categories were showed (p-value < 0.05). Click here for file
