# Supplementary material for: An expression database for roots of the model legume Medicago truncatula under salt stress
Source: BMC Genomics. 2009 Nov 11;10:517. doi: 10.1186/1471-2164-10-517 (PMC2779821; doi:10.1186/1471-2164-10-517)
Supplement: Additional file 1 — Schema of MtED database. Schema of MtED database. [file 1471-2164-10-517-S1.pdf]

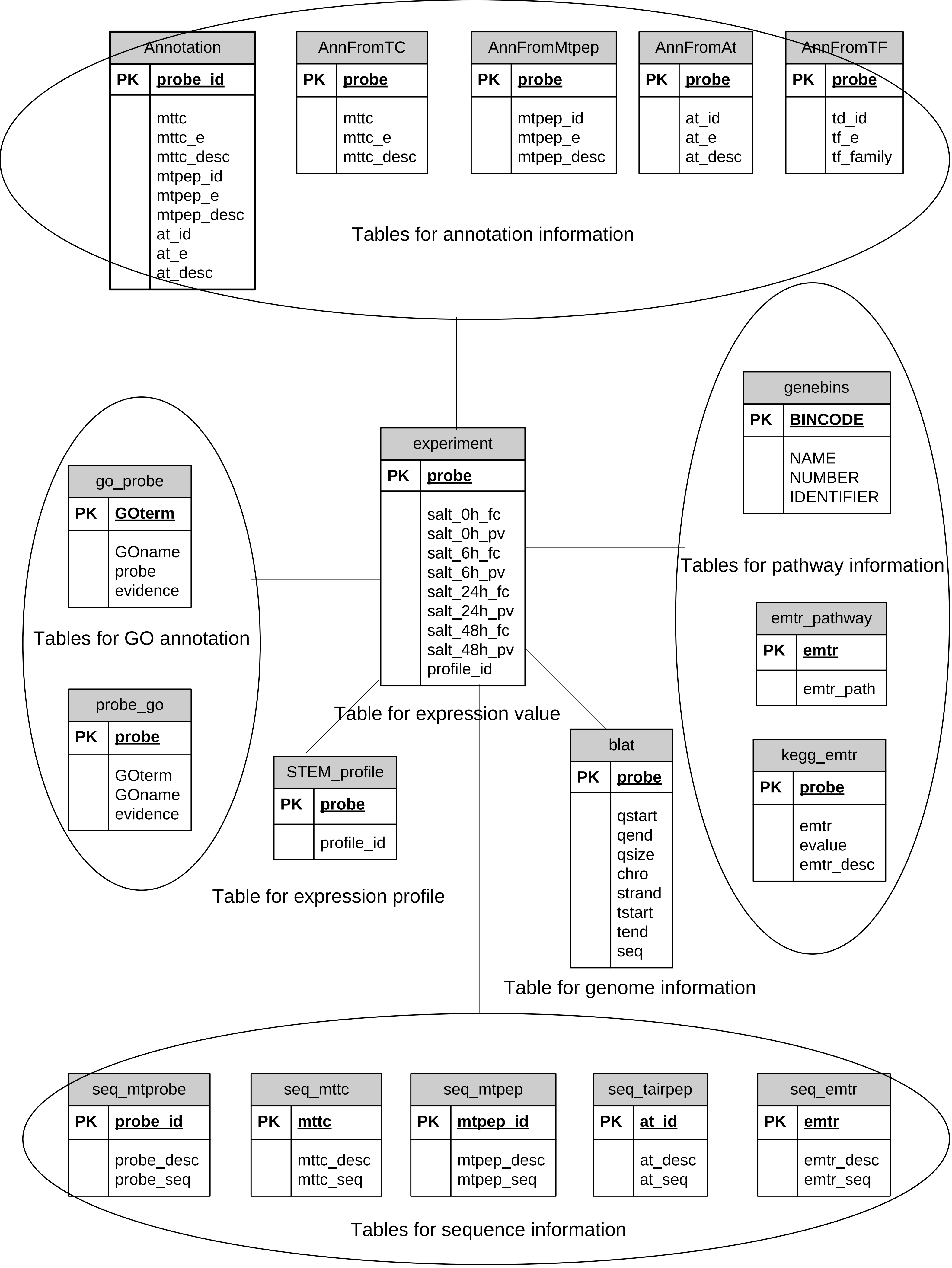

Annotation
PK probe_id
mttc
mttc_e
mttc_desc
mtpep_id
mtpep_e
mtpep_desc
at_id
at_e
at_desc
AnnFromTC
PK probe
mttc
mttc_e
mttc_desc
AnnFromMtpep
PK probe
mtpep_id
mtpep_e
mtpep_desc
AnnFromAt
PK probe
at_id
at_e
at_desc
AnnFromTF
PK probe
td_id
tf_e
tf_family
Tables for annotation information
genebins
PK BINCODE
NAME
NUMBER
IDENTIFIER
go_probe
PK GOterm
GOname
probe
evidence
experiment
PK probe
salt_0h_fc
salt_0h_pv
salt_6h_fc
salt_6h_pv
salt_24h_fc
salt_24h_pv
salt_48h_fc
salt_48h_pv
profile_id
Tables for pathway information
Tables for GO annotation
emtr_pathway
PK emtr
emtr_path
Table for expression value
probe_go
PK probe
GOterm
GOname
evidence
STEM_profile
PK probe
profile_id
blat
PK probe
qstart
qend
qsize
chro
strand
tstart
tend
seq
kegg_emtr
PK probe
emtr
evalue
emtr_desc
Table for expression profile
Table for genome information
seq_mtprobe
PK probe_id
probe_desc
probe_seq
seq_mttc
PK mttc
mttc_desc
mttc_seq
seq_mtpep
PK mtpep_id
mtpep_desc
mtpep_seq
seq_tairpep
PK at_id
at_desc
at_seq
seq_emtr
PK emtr
emtr_desc
emtr_seq
Tables for sequence information
