# Supplementary figures and images for: An expression database for roots of the model legume Medicago truncatula under salt stress
Source: BMC Genomics. 2009 Nov 11;10:517. doi: 10.1186/1471-2164-10-517 (PMC2779821; doi:10.1186/1471-2164-10-517)

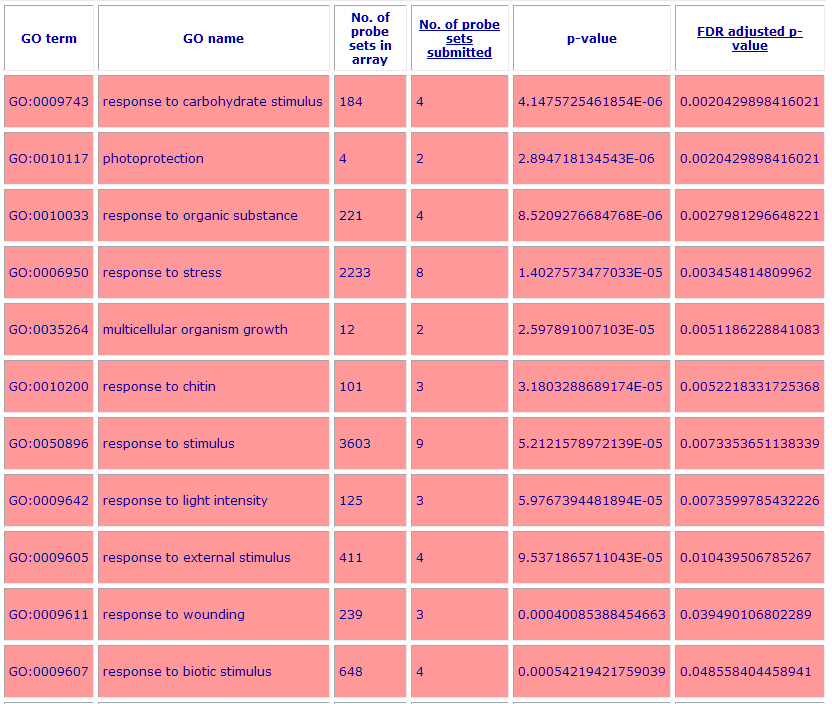

Supplement: Additional file 2 — GO functional category analysis of probe sets contained in "6 h", "24 h", "48 h" and "recovery" simultaneously. Category was sorted by FDR-adjusted P-value. Only significant categories were showed (p-value < 0.05). [file 1471-2164-10-517-S2.jpeg]
